# Improving membrane based multiplex immunoassays for semi-quantitative detection of multiple cytokines in a single sample

**DOI:** 10.1186/1472-6750-14-63

**Published:** 2014-07-15

**Authors:** Raffaele Altara, Marco Manca, Marleen HM Hessel, Ben J Janssen, Harry H A Struijker-Boudier, Rob JJ Hermans, W Matthijs Blankesteijn

**Affiliations:** 1 Department of Pharmacology, Cardiovascular Research Institute Maastricht (CARIM), Maastricht University, Maastricht, The Netherlands; 2 Department of Pathology, Cardiovascular Research Institute Maastricht (CARIM), Maastricht University, Maastricht, The Netherlands

**Keywords:** Cytokines, Chemokines, Multiplex assay, Planar assay, Bead-based assay, Inflammatory mediators

## Abstract

**Background:**

Inflammatory mediators can serve as biomarkers for the monitoring of the disease progression or prognosis in many conditions. In the present study we introduce an adaptation of a membrane-based technique in which the level of up to 40 cytokines and chemokines can be determined in both human and rodent blood in a semi-quantitative way. The planar assay was modified using the LI-COR (R) detection system (fluorescence based) rather than chemiluminescence and semi-quantitative outcomes were achieved by normalizing the outcomes using the automated exposure settings of the Odyssey readout device. The results were compared to the gold standard assay, namely ELISA.

**Results:**

The improved planar assay allowed the detection of a considerably higher number of analytes (n = 30 and n = 5 for fluorescent and chemiluminescent detection, respectively). The improved planar method showed high sensitivity up to 17 pg/ml and a linear correlation of the normalized fluorescence intensity with the results from the ELISA (r = 0.91).

**Conclusions:**

The results show that the membrane-based technique is a semi-quantitative assay that correlates satisfactorily to the gold standard when enhanced by the use of fluorescence and subsequent semi-quantitative analysis. This promising technique can be used to investigate inflammatory profiles in multiple conditions, particularly in studies with constraints in sample sizes and/or budget.

## Background

There is a large body of evidence supporting the use of inflammatory mediators as putative biomarkers for the early detection of a variety of diseases such as heart failure, cancer and infections [1-3]. Hence, determining changes in the levels of inflammatory markers in patients using multimarker-based strategies is expected to lead to better care [4].

In the last decade, multiplex cytokine arrays have become available in a bead-based (or suspension) format showing advantages over the more conventional immunoassays that rely on a solid support [5]. The principle of this assay is quite similar to that of the classic ELISA [6], but the primary antibodies are conjugated to color-coded micro beads rather than ELISA plates and the secondary antibody is attached to a fluorophore. The detection is based on dual-laser flow-based reading where a special instrument classifies the beads and subsequently determines the intensity of the fluorophore, the latter reflecting the concentration of the analytes bound to the beads. Notwithstanding the beauty of the bead-based technology, cases of a loss of sensitivity have been reported [7], resulting in certain proteins becoming undetectable in blood.

An alternative method for simultaneous detection and quantification is a planar assay known as fluorescence-linked immunosorbent assay (FLISA). This assay differs from the ELISA, in that the secondary antibodies are biotin-labeled and the detection relies on the signal intensities emitted by the fluorophore labeled anti-biotin reporters. The sensitivity of this assay is higher than a traditional ELISA and it may be enhanced even further using tyramide signal amplification [8]. FLISA detection is usually suitable for concentrations that are normally present in the low pg/mL range, however it is recommended for the detection of limited number of targets as the optimization can be difficult using this format.

In this manuscript we report an improved planar multiplex assay (Proteome Profiler™). The assay is membrane-based and its protocol also resembles the ELISA principle. Primary antibodies are conjugated to a nitrocellulose membrane in confined spots. A sample pre-incubated with a secondary antibody is then added to the membranes. This assay has been marketed by the manufacturer as a qualitative assay; hence, the membranes should be processed according to a classic Western blot protocol and the results provide information of the presence of an analyte rather than about its amount.

In this manuscript, we present the fluorescent readout of the membranes which substantially increases the sensitivity of the procedure. Moreover, the introduction of an automated procedure to normalize the readout from the analytes allowed us to obtain semi-quantitative results. We evaluated the sensitivity and accuracy of the membrane-based immunoassay and compared the results with those obtained by ELISA. Moreover, we showed the applicability of this planar assay to mouse, rat and human samples.

## Methods

### Ethical disclosure

#### Animal research

This study was carried out in accordance with the recommendations in the Guidelines for the Care and Use of Laboratory Animals of the Dutch Institutes of Health. The protocol was approved by the Committee on the Ethics of Animal Experiments of the University of Maastricht. All surgery was performed under isoflurane anesthesia, and all efforts were made to minimize suffering.

#### Human research

The recruitment of healthy volunteers was performed according to the Dutch Medical Ethical Committee (protocol: METC 11-3-056) and in respect of the Declaration of Helsinki. The individuals in this manuscript have given written informed consent to publish these case details.

### Transverse Aortic Constricted (TAC) Mouse

Pressure overload was induced in male C57BL/6 J mice (Harlan Laboratories, Boxmeer, The Netherlands; N = 5) as previously described [9]. Control male C57BL/6 J mice (Sham; N = 5) underwent the same surgical procedure without the actual tightening of the ligature. Animals were sacrificed four weeks after the Sham or TAC operation and blood samples were collected.

### LPS treated rats

Male Wistar rats (N = 5) were treated, under isoflurane anesthesia, with LPS (Sigma-Aldrich) (0.5 mg/kg) intravenously to induce an acute systemic inflammatory response, and were sacrificed after 30 minutes. For the semi-quantitative test serum from the animals was pooled. A total amount of 1000 μL was used in the membrane-based assay, while a total amount of 50 μL of serum per well was analyzed as required by the ELISA manufacturer’s instructions.

### Sample preparation

#### Serum

Blood was sampled from the abdominal aorta during sacrifice. It was then allowed to clot for 4 hours at 4°C before centrifuging for 10 minutes at 2000 g. Serum was removed and immediately aliquoted and stored (for 6 months on average) at < -80°C.

#### Plasma

Blood was sampled from the abdominal aorta during sacrifice. Plasma was collected using EDTA as an anticoagulant. It was stored for 2 hours at 4°C and centrifuged for 10 minutes at approximately 2000 g. Plasma was removed and immediately aliquoted and stored (for 6 months on average) at < -80°C.

### Membrane stripping

A pool of serum obtained from five TAC mice (200 μL for each) was tested according to the original Proteome Profiler™ Antibody Array based on chemiluminescence. Subsequently, stripping of the secondary antibody was performed by rinsing the membranes three times for 10 minutes with MilliQ water at 70°C. Then the new protocol for fluorescence detection, described in this manuscript (see below), was performed on the stripped membranes.

### TNF-α detection via Enzyme Linked Immunosorbent Assay (ELISA)

Rat TNF-α Quantikine ELISA Kit was purchased from R& D Systems, Minneapolis MN, USA. The assay was performed following the manufacturer’s instructions.

### Proteome Profiler™ Antibody Array: membrane-based assay

Mouse, rat and human Cytokine Array Panel A (R& D Systems) were used according to the manufacturer’s instructions (see the Extracellular Factors session on http://www.rndsystems.com/product_detail_objectname_ProteomeProfilerArray.aspx) until step #9 (Incubate overnight at 2 - 8°C on a rocking platform).

The following modifications of the original protocol were made to adapt it for fluorescence read-out:

1. Carefully remove the membranes and place them into individual plastic containers. Gently wash the membranes with 20 mL of Wash Buffer 1× for 10 min on platform shaker. Repeat the procedure 3 times.

2. Discard the washing buffer and rinse the membranes with 20 mL of blocking solution [Odyssey Blocking Buffer (LI-COR Bioscience, Lincoln NE, USA) and PBS 1× - (1:1)] for 15 minutes on a rocking platform.

3. Every membrane is incubated with 5 mL blocking solution pre-mixed with 3.3 μL IRDye® 800CW Streptavidin (pre-diluted 1:100, LI-COR Bioscience) in 50 mL plastic tubes.

4. Tubes are then placed for 1 hr on a rolling bench protected from the light.

5. Wash the membranes 3 times with PBS 1× supplemented with 0.15%-Tween for 10 minutes.

6. Rinse 4 times with PBS 1×.

7. Leave the membranes to dry on paper in a dark place.

8. Scan with the LI-COR Odyssey infrared imaging scanner (Westburg BV, Leusden, The Netherlands).

Resolution: 84 μm

Quality: High

Focus offset: 0.0 mm

Intensity: 5, adjust as necessary. Avoid overexposure of the membrane. White dots (bleaching) should not appear on the final acquisition.

9. Background subtraction was performed by setting the negative controls of each independent membrane to 0.

NB. The Odyssey imaging scanner adjusts the exposure time automatically upon the signal intensity set. It has default scanning presets. The user can choose from 5 settings through the “quality” option: Lowest, Low, Medium, High and Highest. The settings indicated under point 8 are therefore necessary to have the correct setup, allowing normalization of the data later on.

### Standard curve with human recombinant proteins

Recombinant Human IL-1 beta and Recombinant Human CXCL10/IP-10 from R& D Systems (R& D Systems™) were reconstituted according to the manufacturer’s instructions. Following the reconstitution, the analytes were diluted to generate their respective stock solution.

The stock solutions were used to generate the following standard curves:

Concentrations (pg/mL): 1000, 500, 250,125, 62.5, 31.25.

Human IL-1 beta/IL-1 F2 Quantikine ELISA Kit and Human CXCL10/IP-10 Quantikine ELISA Kit (R& D Systems™) were used to quantify the analytes for further comparison with the Proteome Profiler™ outcome.

### Statistical methods/data analysis

#### Mouse/rat data

All data included in this manuscript are expressed in units according to the international system. After background subtraction, polynomial curve fitting was used to infer the calibration curve for each membrane using Prism5 (GraphPad Software, Inc.; La Jolla CA, USA) and R [10]. Linear regression was used to test the co-linearity between ELISA and the membrane-based assay read-outs. The results are illustrated by bar and scatter plots.

#### Human data

Raw data have been imported in R. Background was subtracted from raw data for each membrane. The readouts of the membranes have been tested for a linear relationship with the predicted concentrations by use of the MASS package (‘Modern Applied Statistics with S’ (4th edition, 2002)). Study of the quality of predictions was performed by gvlma (Global Validation of Linear Models Assumptions).

## Results

### Comparison of fluorescence and chemiluminescence detection

**Figure 1:**
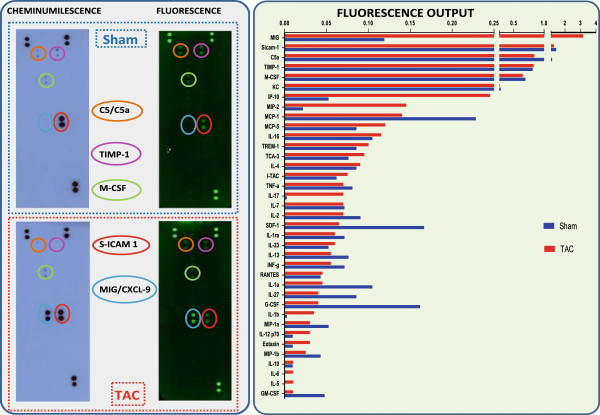
**Comparison of chemiluminescence and fluorescence detection.** In the left panel, chemiluminescence and fluorescence images, obtained from the same membrane after stripping, are represented. The latter method yielded a considerably higher number of detectable analytes that can be visualized (green spots). The fluorescence readout allowed to compare the circulating inflammatory profile of pooled samples (200 μL each) of 5 TAC operated mice to the relative shams (see graph in the right box).

In Figure 1, two membranes that first were developed by chemiluminescence and then re-developed after stripping of the streptavidin-enzyme complex from the biotinylated secondary antibody are shown. The number of spots that could be detected, even visually, appeared to be 6- fold higher following the fluorescent (n = 30) compared to the chemiluminescent (n = 5) detection method. As a result, the membrane-based technique allowed the detection of the robust increase of at least 4 analytes (MIG, IP-10, MIP-2 and IL-17) out of the 30 detectable inflammatory mediators present in pooled serum samples of five TAC operated mice with similar hemodynamic characteristics.

### Comparison of the membrane-based assay with fluorescence detection to ELISA

**Figure 2:**
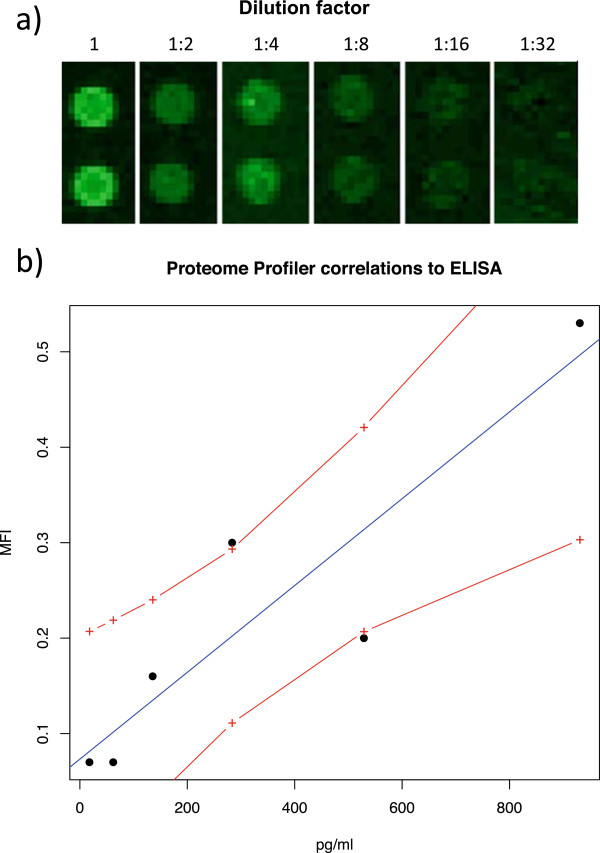
**Membrane-based cytokine arrays as semi-quantitative assay. (a)** TNF-α signal derived from the membrane-based assay for six progressive dilutions of a pool of plasma obtained from two LPS treated rats. The concentrations range from 930 to 18 pg/mL (from left to right). In **b**, the correlation between the membrane-based assay intensity and ELISA concentration detected for the same samples measured with the two different techniques is shown. The red dashed lines delimited the 95% confidence interval.

In order to prove the feasibility of the optimized membrane-based technology as a semi-quantitative assay, biological samples already containing high levels of cytokines were used for validation. For this purpose, a consecutive dilution series of LPS-treated rat blood was created and TNF-α was measured using both the membrane-based assay and ELISA. As shown in Figure 2a, the fluorescence intensity (FI) decreased linearly with increased dilution. Moreover, the correlation of the fluorescence intensity with the serum concentration determined by ELISA was Pearson’s r = 0.91 (Figure 2b).

### Applicability to human cytokines

**Figure 3:**
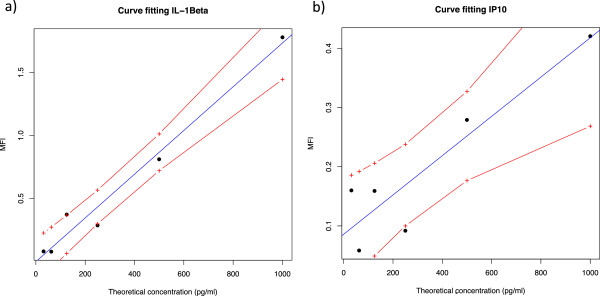
**Curve fitting for IL-1β and IP-10.** Visualization of the regression line (blue) fitted for the theoretical concentrations and membrane’s fluorescence readouts of IL-1β **(a)** and IP-10 **(b)**. The dots are compactly distributed around the predicted line, and the unexplained error is small. The red lines delimited the 95% of CI. The equations of the curves are: **a)** y = 561.73 × + 8.97 with Pearson’s r = 0.97; **b)** y = 1830.6 × – 79.4 with Pearson’s r = 0.91. The red dashed lines delimited the 95% confidence interval.

**Figure 4:**
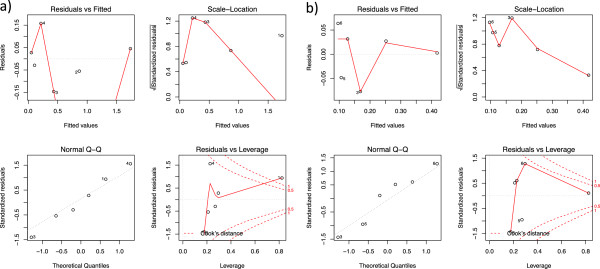
**Model diagnostics of the curve fitting of IL-1β (a) and IP-10 (b).**"Residuals vs. Fitted" and "Scale-Location" show that a trend appears in the residuals distribution for concentrations below 400 ng/mL. The “Q-Q plot” suggests that residuals are not normally distributed. Whilst this does not affect the goodness of the coefficient estimation, it impairs the t-distribution and thus renders the use of p-values meaningless. The Cook’s distance suggests that 2 of the most diluted samples could be considered outliers in their influence on the model fitting. However, Cook’s distance has no unambiguous interpretation and the best decision is to look at the original distribution, where the 2 incriminated points show no indication of anomalous position. The red dashed lines delimited the 95% of CI.

As proof of concept of the translatability of the fluorescent protocol to human research, a series of tests on consecutive dilutions of known cytokines in buffer were assayed. As shown in Figure 3, the Fluorescence Intensity (FI) readout of the membranes was almost co-linear with the predicted concentrations (Pearson’s r value of 0.98 and 0.91 for IL-1β and IP-10, respectively). Testing the model (Predicted concentrations ~ Membrane readouts) fitting to detect biases and to check the overall quality of the distribution also gave promising results (Figure 4). The residuals vs. fitted values appeared to be skewed towards the low values. Overall, these results indicate that the residuals behave randomly, suggesting that the model fits the data well.

## Discussion

**Figure 5:**
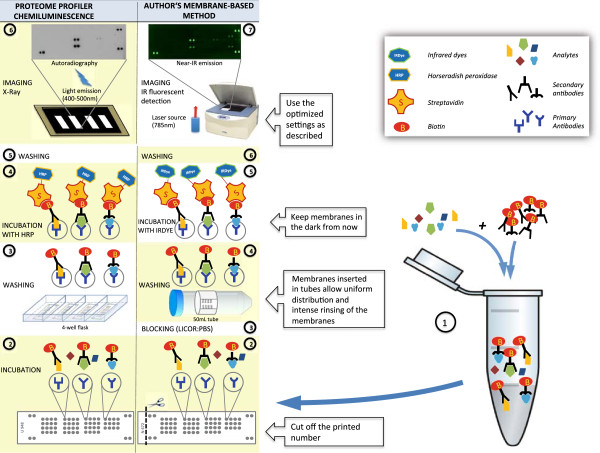
Schematic representation of the main differences between the Proteome Profiler intended for chemiluminescence and the fluorescent detection method and semi-quantitative analysis protocol proposed in this manuscript.

In the present study, we show the superior sensitivity of fluorescence over chemiluminescence detection of a membrane-based assay for inflammatory mediators. Although this assay is intended for qualitative use by the manufacturer, we improved it using a fluorescence reporter system and a near-infrared imaging device to acquire the signal, and showed that the assay could be upgraded to yield semi-quantitative data (see Figure 5). We demonstrate that by normalizing the readouts to the background reference spots present on each membrane and using specific instrument settings the readout becomes semi-quantitative and shows a strong correlation with the outcomes of ELISA. Our adaptations make the membrane-based analysis suitable for use in situations where the amount of sample is limited, as information of ~30 analytes could be obtained from only 1 mL of serum.

The improved membrane-based technique allowed the screening and detection of a large number of circulating inflammatory mediators in an in-vivo model for circulating cytokines/chemokines, which would be limited in conventional ELISAs due to sample availability and/or cost. Indeed, small animal models are known to yield low sample volumes, so to perform multiple ELISAs a large amount of animals would be necessary. Besides, to perform up to 40 classical ELISA investigations it would be rather laborious and costly.

In this study, a model of cardiovascular disease has been used to test the screening performance of the improved membrane-based assay on pooled samples obtained from mice. The TAC mouse model used is actually a known model for cardiac hypertrophy and it is conclusively proved that inflammation becomes activated [11,12] during cardiac remodeling. Moreover, the cardiac inflammatory mediators are released into the circulation [13], but the complete list of all the inflammatory mediators that are circulating in blood is still far from complete. In our investigation we obtained evidence that novel analytes (i.e. MIG and IP-10) can be found in the circulation of mice exposed to TAC for 4 weeks, giving to us a starting point for future investigations. However, our experimental approach (chemiluminescent detection- stripping –fluorescent detection) may have affected the actual inflammatory profile. Indeed, the efficiency of the stripping and the protein loss during that passage could not be assessed. These results therefore need to be interpreted with caution, as the observed inflammatory profile may not reflect the actual one that is present in mice after 4 weeks of TAC.In this study we are the first to introduce the versatility of the membrane-based assay, commercially known as Proteome Profiler, as a multiplex detecting assay for semi-quantitative analysis of inflammatory mediators. The use of the fluorescence detection protocol allowed us to accurately read the intensity of the spots at standardized exposure times (see materials and methods) which in turn allowed us to create a normalization protocol. The distinct advantages were that the measurements could be repeated without variation (data not shown) and the reading outcomes were comparable among the membranes, as the “development procedure” remained the same. This approach favored the construction of concentration curves with satisfactory results when it came to linearity (Figures 2 and 3).

One of the reasons that drove us to implement the membrane-based technique was to test whether it was possible to avoid the “hook effect”. This effect is a phenomenon that can occur in multiplex immune-assays when the level of the analyte of interest is close to the lower detection limit; hence the signal to noise ratio is too low and the readouts corrected for the dilution factor result in an overestimation of the actual concentration. In previous experiments performed using the multiplex bead-based technology, we ran into such artifacts in several cases (data not shown). We observed that the detection of many inflammatory mediators in rodent blood was impaired and therefore this approach was only useful for the detection of analytes with high circulating levels.To test the performance of our adapted membrane-based assay at low analyte concentrations, we tested our model (Predicted concentrations ~ Membrane readouts) fitting to detect biases and to check the overall quality of the distribution also at very low side of the curve (Figure 4). Below 200 pg/mL the residuals diverged, possibly showing a technical limitation of the membrane based assay. In fact, the residuals did not show any co-linearity with the fitted values, and the residuals versus leverage plots showed a well-behaved test. This behavior suggests that there is room for technical development to improve the reliability of the assay at low concentrations.

## Conclusion

We present the potential use of a planar method as multiplex assay for the identification of a large panel of inflammatory mediators in blood. By switching from the chemiluminescent to the fluorescent readout and adapting our detection protocol, we showed that a membrane-based assay can yield semi-quantitative results for the detection of multiple cytokines and chemokines in mouse, rat and human blood samples. Applying this novel promising technique will allow scientists from several fields to extend their investigations in a prominent way, particularly when an inventory of multiple inflammatory markers is needed in a setting of low sample sizes and/or budget constraints. Considering the outcome of this study and what we known from the commercially available kits for cytokine/chemokine determination, we present a descriptive table that summarizes the major pros and cons of the assay with the respect to the two commonly alternative methods (see Additional file 1: Table S1).

## Competing interests

The authors declare that they have no competing interests.

## Authors’ contributions

RA conceived the design of the study, carried out the immunoassays and part of the animal work and drafted the manuscript. MM participated in the design of the study and performed the statistical analysis. MHMH carried out the animal work and contributed with acquisition and interpretation of the data. BJJ participated in the design of the study and coordinated the animal work. HAJSB participated in the study design and coordination and critically revised the manuscript. RJJH conceived the design of the study, coordinated the project and critically revised the manuscript. WMB participated in the design of the study, coordinated the project and critically revised the manuscript. All authors read and approved the final manuscript.

## Supplementary Material

Additional file 1: Table S1 Advantages and disadvantages of the existing immunoassays kits for cytokines/chemokines determination. Click here for file

## References

[B1] Motilva V Inflammation and cancer: new targets and novel therapeutic approach Curr Pharm Des 2012 18 26 3829 3830 22804332 10.2174/138161212802083671

[B2] Corsten MF Schroen B Heymans S Inflammation in viral myocarditis: friend or foe? Trends Mol Med 2012 18 7 426 437 22726657 10.1016/j.molmed.2012.05.005

[B3] Lam HS Ng PC Biochemical markers of neonatal sepsis Pathology 2008 40 2 141 148 18203036 10.1080/00313020701813735

[B4] Ahmad T Fiuzat M Felker GM O’Connor C Novel biomarkers in chronic heart failure Nat Rev Cardiol 2012 9 6 347 359 22450126 10.1038/nrcardio.2012.37

[B5] Leng SX McElhaney JE Walston JD Xie D Fedarko NS Kuchel GA ELISA and multiplex technologies for cytokine measurement in inflammation and aging research J Gerontol A Biol Sci Med Sci 2008 63 8 879 884 18772478 10.1093/gerona/63.8.879 PMC2562869

[B6] Lequin RM Enzyme immunoassay (EIA)/enzyme-linked immunosorbent assay (ELISA) Clin Chem 2005 51 12 2415 2418 16179424 10.1373/clinchem.2005.051532

[B7] Liu MY Xydakis AM Hoogeveen RC Jones PH Smith EO Nelson KW Ballantyne CM Multiplexed analysis of biomarkers related to obesity and the metabolic syndrome in human plasma, using the Luminex-100 system Clin Chem 2005 51 7 1102 1109 15976097 10.1373/clinchem.2004.047084

[B8] Haab BB Zhou H Multiplexed protein analysis using spotted antibody microarrays Methods Mol Biol 2004 264 33 45 15020778 10.1385/1-59259-759-9:033

[B9] van Eickels M Grohe C Cleutjens JP Janssen BJ Wellens HJ Doevendans PA 17beta-estradiol attenuates the development of pressure-overload hypertrophy Circulation 2001 104 12 1419 1423 11560859 10.1161/hc3601.095577

[B10] Wass JA R: a language and environment for statistical computing R Found Stat 2012 Version 2.11.1 (2010-05-31) 1

[B11] Christia P Bujak M Gonzalez-Quesada C Chen W Dobaczewski M Reddy A Frangogiannis NG Systematic characterization of myocardial inflammation, repair, and remodeling in a mouse model of reperfused myocardial infarction J Histochem Cytochem 2013 61 8 555 570 23714783 10.1369/0022155413493912 PMC3724390

[B12] Coggins M Rosenzweig A The fire within: cardiac inflammatory signaling in health and disease Circ Res 2012 110 1 116 125 22223209 10.1161/CIRCRESAHA.111.243196

[B13] Braunwald E Biomarkers in heart failure N Engl J Med 2008 358 20 2148 2159 18480207 10.1056/NEJMra0800239

