# Supplementary material for: Improving membrane based multiplex immunoassays for semi-quantitative detection of multiple cytokines in a single sample
Source: BMC Biotechnol. 2014 Jul 15;14:63. doi: 10.1186/1472-6750-14-63 (PMC4107595; doi:10.1186/1472-6750-14-63)
Supplement: Additional file 1: Table S1 — Advantages and disadvantages of the existing immunoassays kits for cytokines/chemokines determination. [file 1472-6750-14-63-S1.doc]

**Additional file 1: Table S1.** **Advantages and disadvantages of the existing immunoassays kits for cytokines/chemokines determination**

|  | **ELISA** | | **Membrane-based assay** | | **Beads-based assay** | |
| --- | --- | --- | --- | --- | --- | --- |
| N. of Analytes Determined | 1 / well | | Up to 40 / membrane | | Up to 500 / well | |
|  | One analyte per plate | | All analytes on one membrane | | All analytes in one well | The panel has to be combined: not all analytes can be measured together |
| Sample Size Needed (μL) | 50-100/ well | | 500-1000/ membrane | | 25-50/ well | |
|  | High volume requested for multiple analytes | | Low sample size considering the number of analytes determined | | Very low sample size | |
| Specific devices | Plate reader | | Infrared imaging system | | Luminex™ plate reader + magnetic or standard washer | |
|  | Basic, simple to use, low budget | | Easy to use, multitasking (i.e. WB imaging) | Intermediate cost | Sophisticated, expensive, dedicated to beads-based assay only | |
| Range of concentrations | Intermediate | | Large dynamic range | | Dynamic range | |
| Handling skills | Intermediate | | Novice/Intermediate | | Expert | |
| Cost of the commercial kit | Various costs | Expensive when requested for multiple analytes determination | Low budget considering the number of analytes | | Low budget when compared to a single ELISA kit | Expensive |
| Time (hrs) | 5 – O.N. | | 5 – O.N. | | 6 – O.N. | |
| Other | May need multiple dilutions | | Ability to perform repeated measurements.  Reduces matrix effect | | Ability to perform repeated measurements | Multiple dilutions may apply. Matrix effect probability |
| Assessment | Quantitative | | Semi-quantitative | | Quantitative and qualitative | |

The pros and cons are described in the green and orange columns, respectively.
